# Data on regional availability and accessibility of abortion providers in Germany

**DOI:** 10.1007/s43999-023-00036-4

**Published:** 2023-12-15

**Authors:** Rona Torenz, Heike Vollmer, Sarah Eckardt, Anke Wyrobisch-Krüger, Ines Thonke, Daphne Hahn

**Affiliations:** Department of Health Sciences, Hochschule Fulda–University of Applied Sciences, Fulda, Germany

**Keywords:** Abortion, Healthcare variations, Access, Accessibility, Availability, Regional variations

## Abstract

Since 2003, the number of facilities reporting abortions to the Federal Statistical Office has decreased by 46% from 2050 to 1092 in 2021. In the last 5 years, the decrease slowed down. The media report that there is a shortage of physicians willing to perform abortions in some regions of Germany. Reduced availability and accessibility of providers are barriers to access that can negatively impact the health and well-being of abortion seekers. To date, there is no scientific evidence on regional differences in access to abortion in Germany. The article answers the following questions: What data are available in Germany regarding the availability and accessibility of abortion providers? How informative is this data? To what extent do the data show regional differences in availability and accessibility? We conclude that the available data are not sufficient to adequately describe regional variations in the provision of abortion care services. Nevertheless, they give clear indications of differences regarding provider density, provider workload and spatial accessibility, especially between the northern and eastern states on the one hand and the southern and western states on the other. We describe needs and policy recommendations for adequate data collection.

## Introduction

In recent years, there have been an increasing number of media reports noting a regional shortage of doctors offering abortions - this applies to Lower Saxony, Lower Bavaria and the regions of Flensburg, Passau and Trier, among others. Professionals, such as psychosocial counsellors, and affected women^1^ describe difficulties in finding doctors, getting the necessary short-term-appointments, or the need to travel long distances [1–4]. According to the Federal Statistical Office of Germany (StBA), the number of facilities reporting abortions to the StBA has decreased by 46.7% from 2050 in 2003 to 1092 in 2021 [5]. From 2018 to 2021, the number of reporting facilities decreased by 5,9% from 1160 to 1092 and increased slightly to 1105 in 2022 (figures refer to the 4th quarter of the year in each case). Federal health reporting has also addressed the topic of abortion in recent years [6, 7].

### Abortion as a component of reproductive health

The current WHO guideline on abortion care recommends guaranteeing access to abortion to ensure reproductive health. In this framework, access is related to time and space, such as the ability to schedule an appointment promptly and to reach a clinic nearby one’s home [8]. In its resolution of 24 June 2021, the Council of Europe states that practical barriers in access to abortion can jeopardize the assurance of reproductive and sexual health [9].

Low availability and spatial accessibility of abortion providers can result in longer waiting times, longer travel distances, higher costs, more organizational effort, and experiences of stigma due to forced disclosure, among other consequences [10–12]. These consequences represent barriers to access and particularly affect vulnerable abortion seekers, for example, those with lower socioeconomic resources, language barriers, violent partners, or disabilities, as well as minors and undocumented persons [13, 14]. Barriers to access can negatively impact health and well-being. Delays in seeking care and subsequent later termination of pregnancy can potentially lead to greater distress and higher medical risks [8]. They may also prevent access to medical abortion, as this is only possible up to the completed 7th week of pregnancy (p.m.) in Germany, thus limiting the free choice of method [15]. In addition to the desire for secrecy or privacy, the costs associated with abortion and the long distance to the nearest abortion provider are common reasons why over 1000 people who did not intend to be pregnant in Germany have chosen abortion outside of regular care through Women on Web since 2019 [14].

Therefore, access to safe abortion is essential for the realization of reproductive health. In their concept of access to health care, Penchansky and Thomas defined five dimensions that mutually interact to generate access: Availability, Accessibility, Responsiveness, Affordability, and Acceptability [16]. The present analysis focuses on two of those five dimensions: Availability refers to the actual physical availability of services. It is defined as the relationship between the capacity of existing services and the number and needs of clients [16]. Accessibility includes the relationship between the location of the service and the location of the clients, where the necessary travel time, distance and mobility of the clients are also important [16]. Especially with the tight legal time limit and the higher medical risk the more advanced the pregnancy, accessibility of abortion providers in terms of time and space is essential [17].

### Legal conditions in Germany

#### The unlawfulness of abortion and consequences for the data basis

In Germany, abortions are regulated in §§ 218 ff. of the German Penal Code (StGB). In 2021, 95,8% of all abortions in Germany took place after mandatory counselling (§ 218a para. 1 StGB) [18]. This means that an abortion seeker can have a pregnancy terminated by a doctor up to 12 weeks after conception, following mandatory counselling (“Pflichtberatung”) at a state-approved counselling centre and a three-day waiting period. The introduction of mandatory counselling took place in 1995. Before reunification, women in East Germany could terminate a pregnancy within the first 12 weeks on their own request [19]. Special departments existed in almost all hospitals in the German Democratic Republic (GDR), which performed the procedure frequently and routinely [19]. In the Federal Republic of Germany (FRG), abortions were illegal under § 218 StGB, but possible under certain exceptions. In this context, guidelines and legal practice were more restrictive in the southern federal states than in the northern federal states [20, 21]. For example, in Baden-Wuerttemberg and Bavaria only hospitals were allowed to perform abortions [20, 21]. The more restrictive regulations in the southern federal states resulted in many women travelling to the northern federal states to have abortions [21]. After reunification, a harmonization of the legal conditions in East and West became necessary. The compromise agreed upon by the German Parliament in 1992, which permitted legal abortion during the first 12 weeks after mandatory counselling, was declared unconstitutional by the Federal Constitutional Court (BVerfG) in 1993. The ruling determined that abortions after mandatory counselling could not be considered legal, even if they were allowed to remain unpunished (§ 218a StGB).

The stipulation by the BVerfG, that the lawfulness of abortions after mandatory counselling cannot be established, led to the ruling that they have to be excluded from regular services provided by statutory health insurance funds (SHI) [22]. According to § 24b of the German Social Code, Book V, only abortions that are not unlawful, i.e. those with a medical or criminological indication, are included among the SHI-funded services. According to § 19 of the Act on Assistance to Avoid and Cope with Conflicts in Pregnancy (SchKG), women with low income can apply to a health insurance fund office to have the costs covered. In such cases, the costs are reimbursed by the social security funds of the federal states; only the application process and the billing are administered by the health insurance funds. Thus, abortions are also not part of the regular planning of healthcare needs of SHI-accredited physicians.

Instead, according to § 13 para. 2 SchKG, the states must ensure a “sufficient” supply of abortion facilities. While the number of pregnancy counselling centres is regulated and § 4 para. 1 SchKG specifies a metric of one full-time position per 40,000 inhabitants, there is no precise definition of what constitutes a “sufficient” number of abortion providers. In the 1993 ruling of the BVerfG, it was defined that provision is sufficient if the woman needs a maximum of 1 day to travel to and from the facility to perform the abortion [22].

The regular hospital planning of the federal states also does not guarantee the coverage of clinics that perform abortions, as they are not part of the services that are regularly covered by hospitals. In at least two German states, abortions were included in hospital planning laws as part of the services to be provided, but they are currently not included in any state law.^2^

#### Federal statistics and reporting obligations

According to §§ 15 ff. of the SchKG, the Federal Statistical Office is legally bound for conducting federal statistics on abortions. For this purpose, the StBA collects, among other things, the number of abortions performed, the legal grounds for the abortion (mandatory counselling or indications), the federal state in which the abortion was performed, and the federal state in which the pregnant woman resided. Facilities in which abortions were performed within the last 2 years are required to provide information to the StBA according to § 18 para. 1 SchKG. These providers are referred to as “reporting facilities.” The StBA records the names and addresses of the reporting facilities according to § 17 para. 1 SchKG as so-called auxiliary data, which may only be collected for the technical realization of the statistics and are therefore not made available for scientific evaluations. A consequence of this set of rules is that addresses and exact locations of reporting facilities are available, but nobody is allowed to evaluate the data. According to Section 18 (3) SchKG, in the case of medical practices, the state medical associations and, in the case of hospitals, the responsible health authorities are to provide the StBA with the addresses of the facilities that perform abortions upon request.

#### List of the German Medical Association and research questions

With the “Act to Improve Information on Abortion” in March 2019, the German Medical Association was commissioned under § 13 para. 3 SchKG to establish a list of facilities that perform abortions, which is to be updated on a monthly basis. The list contains the name, address and, if available, information on the methods of abortion offered. Inclusion on the list is by voluntary notification by the facilities. It is published online on the websites of the German Medical Association and the Federal Centre for Health Education (BZgA).

This paper is part of the ELSA research project [23].^3^ The main goal is to assess the regional availability and accessibility of abortion providers in Germany. Under the legal conditions described, the question of which data is available for such a study is of the utmost importance. The article therefore answers the following questions:

What data are available in Germany regarding the availability and accessibility of abortion providers? How informative is this data? What findings result from the evaluation of suitable and available data for the regional availability and accessibility of providers in Germany?

Since answering the first two questions is important for the methodological approach taken to answer the third question, this article has an unusual structure: first, we will present the results of the first two questions. Based on these data, we will present the methodological approach for answering the third question.

## Results

The results are presented in two subsections: in the first subsection, we examine which data are available at all on the basis of the legal framework described above. We assess these data regarding how informative this data is regarding regional availability and accessibility of providers. In the second subsection, we analyse selected data with regard to regional differences in availability and accessibility. A description of the methodological procedure for analysing the data follows this subsection.

### Informational value of available data

#### List of the German Medical Association

Due to the voluntary notification of the facilities, the information in the list of the German Medical Association is highly incomplete and unsuitable for a comprehensive representation of abortion providers in Germany. As of June 5th, 2023, there are only 365 addresses on the list [24], while the StBA indicates 1092 reporting facilities in the 4th quarter of 2021 [25].

#### Available data in the federal states

In the federal states, the available data on abortion providers differ. In Lower Saxony, physicians who perform abortions are recorded via registration with the state medical association [26], and in Berlin, Bremen, and Hamburg, via a list maintained by the respective authority [27–29]. In Brandenburg, Saxony-Anhalt, Rhineland-Palatinate, and Bavaria, ambulatory facilities must apply for a special permit from an authority to perform abortions [30–33]. Therefore, data on the permit holders are available in these states. However, the possession of a permit does not mean that a physician actually provides abortions. In Hesse, a list was maintained from 2015 to 2018 based on billing data for abortions with reimbursement according to § 22 SchKG. However, with reference to the fact that the StBA publishes the number of reporting facilities in the federal states, this list has been discontinued since 2019 [34]. In the remaining federal states, there is no separate data collection. Due to the lack of a similar data collection in all federal states, no comprehensive statements on the regional distribution of abortion providers are possible on the grounds of data collected by the federal states.

#### Billing data of the health insurance funds

Under the given legal conditions, there is no recording of abortions after mandatory counselling via the regular billing data of the health insurance funds. An inquiry at the BARMER Institute for Health System Research (bifg) also revealed that the data available there on physicians who bill for abortions upon assumption of costs by the states is also not comprehensively recorded and that there are obviously large regional gaps here (BARMER Institute for Health System Research (bifg), personal communication, 21.06.2022). Since lawful abortions with a medical or criminological indication are part of the regular health insurance benefits, the billing data would be complete here. However, because the number of these abortions is so low, the related billing data would only cover a small fraction of the doctors who perform abortions.

#### Federal statistics

Since the 4th quarter of 2018, the StBA is publishing the number of reporting facilities in the federal states online [5]. The informative value of the StBA figures on reporting facilities is limited in terms of regional availability and accessibility: Firstly, it is not apparent how many physicians offer abortions at this reporting facility and whether some physicians perform abortions at multiple reporting facilities. Consequently, the number of reporting facilities does not provide a clear indication of the number of providers. Secondly, the number of reporting facilities says nothing about the extent to which they make it public that they perform abortions, i.e., whether unintended pregnant women can access the facility at all. Thirdly, the figures do not indicate how much capacity the facilities have available for carrying out abortions. A reporting facility can be a big university hospital or family planning centre carrying out a high number of abortions per year or a medical practice of one doctor performing abortions only for own patients in exceptional circumstances. Fourthly, the StBA provides the figures of the reporting facilities only on the level of the federal states, because only the federal state is allowed to be collected according to § 16 para. 1 No. 6 SchKG. The level of the federal states, however, does not represent a sufficient degree of detail for the assessment of the regional situation regarding the provision of abortion care. By way of comparison: In the needs-based planning, which is used for the distribution of SHI-accredited physicians in the region in relation to the population, different planning levels are used depending on the type of medical service. For gynaecological care, the district-free city, the county or the county region are designated as planning areas [35].^4^ Even specialized medical care (e.g. radiologists or child and adolescent psychotherapists) is planned at the level of spatial planning regions, which usually combine about four counties and are thus below the federal state level.

The overall picture shows that there is currently no comprehensive, complete and up-to-date list of abortion providers in Germany, nor can it be compiled from the available data. Of all the available information, the data from the StBA on the reporting facilities is the best option for creating a nationwide overview of the regional distribution of providers, although this is associated with the limitations described above.

### Evaluation of available data

#### Methods

For this subsection, we use data from the annual statistics on abortions by the Federal Statistical Office (StBA) for the year 2021 [18]. These are the data on the number of reporting facilities, abortions, and abortion rates (number of abortions per 10,000 women) in the different federal states. In addition, we use data from a special evaluation of the StBA commissioned by the ELSA research project [36]. This special evaluation includes a differentiation of the reporting facilities in the federal states according to the number of abortions they reported in 2020. For this purpose, the number of reported abortions was subdivided into the size classes 0,^5^ 1–10, 11–50, 51–250, 251–500, 501–1000, and 1001 and more. For data protection reasons, the StBA combined several size classes in some states. The special evaluation was commissioned by ELSA in order to be able to map the distribution of abortions among the reporting facilities.

In addition to the provided data from the Federal Statistical Office, we use figures from two parliamentary inquiries in the Bavarian and Hessian state parliaments. For the seven Bavarian administrative districts, the number of ambulatory facilities licensed^6^ to perform abortions and the number of clinics willing to perform abortions after mandatory counselling are available for 2020 [33]. For Hesse, the number of facilities performing abortions is available at the county level for 2018 [37]. This number of facilities (*n* = 114) differs from the number of reporting facilities recorded by the StBA for Hesse in 2018 (*n* = 81) [38], as the Hesse state government data is not based on reporting facilities, but on billing data for reimbursement [34].

We evaluate the described data with respect to three different indicators that relate to the access dimensions of availability and accessibility. In order to obtain clues about regional differences in the availability of providers, we first calculate the ratio between women of reproductive age (15–49 years) [39] and reporting facilities or, for Hesse and Bavaria, abortion providers (indicator “provider density”). Second, we calculate the average distribution of abortions among reporting facilities for the states and differentiate this distribution by reporting facilities of different sizes (indicator “provider workload”). Third, for insight into regional differences in spatial accessibility, we calculate the ratio of reporting facilities/abortion providers to area (indicator “spatial accessibility”).

#### Provider density

**Fig. 1:**
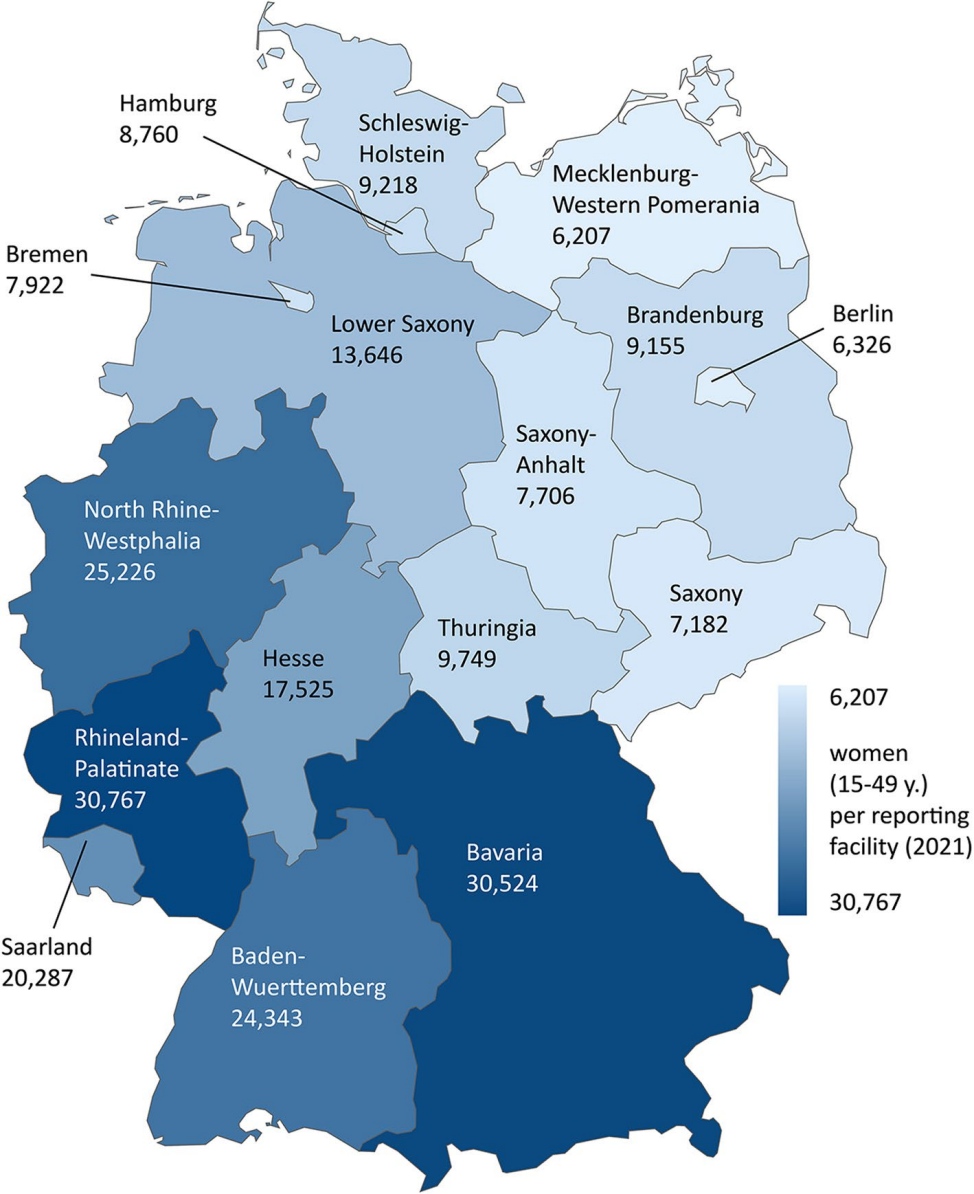
Provider density in Germany (2021)

The data show big differences in provider density between federal states: In 2021, there were between 6207 (Mecklenburg-Western Pomerania) and 30,767 (Rhineland-Palatinate) women of reproductive age per reporting facility in Germany (Fig. 1). The map shows a clear gradient: the eastern and northern states all have higher provider densities – e.g. lower numbers of women per facility – than the western and southern states.

**Fig. 2:**
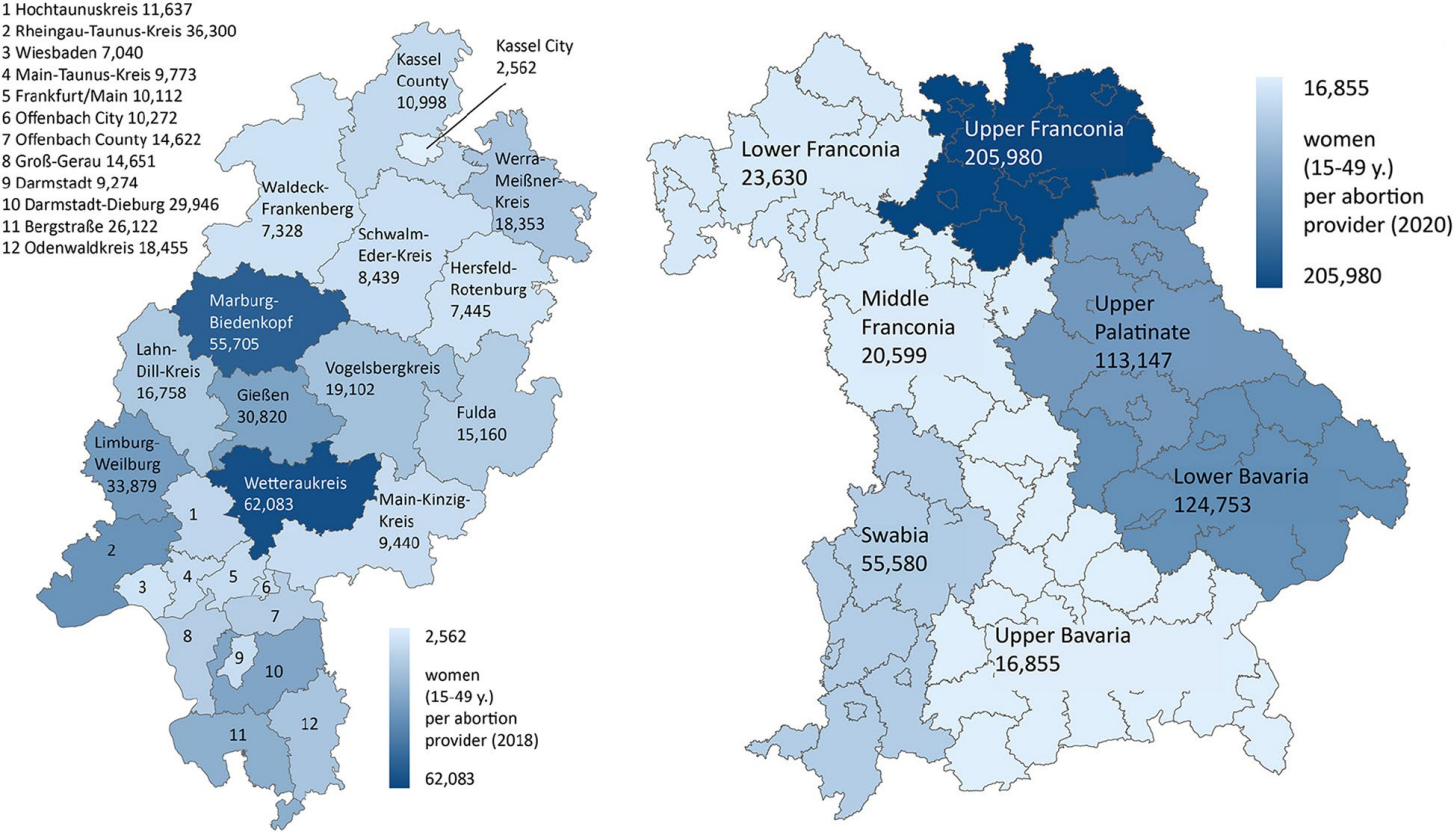
Provider density by county in Hesse (2018, left) and by administrative district in Bavaria (2020, right)

Evaluation of the data for provider density in Bavaria and Hesse at administrative district resp. county level shows that the figures can differ even more within a federal state (Fig. 2). Here, the figures in Hesse ranged from 2562 (Kassel City) to 62,083 (Wetteraukreis) and in Bavaria from 16,855 (Upper Bavaria with Munich) to 205,980 (Upper Franconia).

#### Provider workload

The total number of reported abortions has gone down from 128,030 to 94,596 abortions per year from 2003 to 2021, a decrease of 26,1% [18]. Proportionally, there has also been a decrease from 65 to 56 abortions per 10,000 women aged 15–49 years, a decrease of 13,8% [18]. Over the same period, the number of reporting facilities has fallen by a total of 46,7%, from approximately 2050 in 2003 [5] to 1092 in the 4th quarter of 2021 [25]. Thus, the average number of abortions per reporting facility has increased from 62 to 86 and thus by 27,9%. In the last 5 years, the provider workload rose from 87 abortions per reporting facility in 2018 to 90 in 2020, but then fell back to 87 in 2021. There are currently different developments in the federal states: For example, Saarland recorded from 2018 to 2021 an increase in provider workload by 63 abortions per reporting facility, Bremen a decrease by 57 and Rhineland-Palatinate also a decrease by 25.

**Fig. 3:**
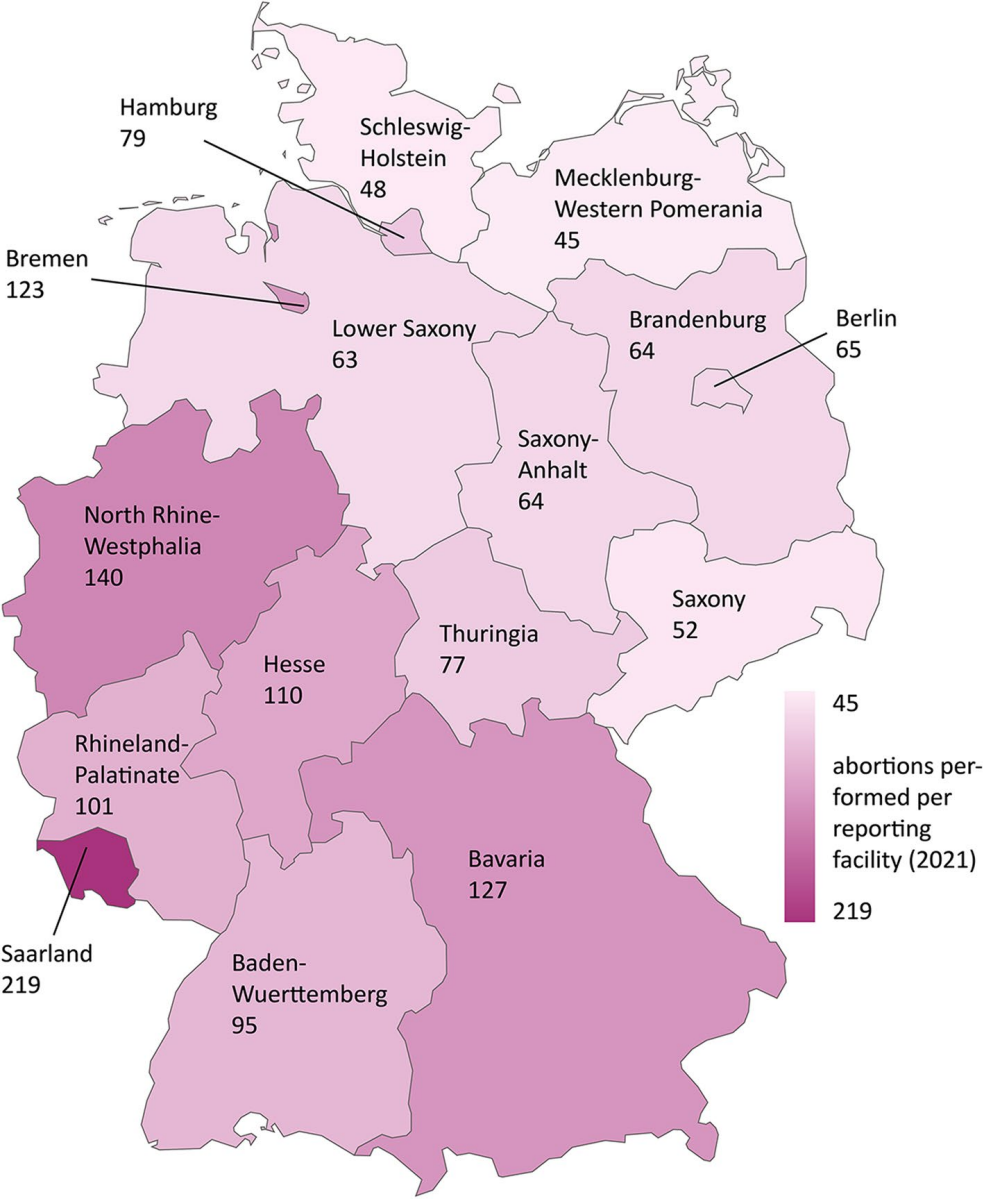
Abortions performed per reporting facility in Germany (2021)

For the year 2021 a comparison of the figures for the individual federal states shows large differences: While in Saarland in 2021 there are on average 219 abortions per reporting facility, this figure is 45 in Mecklenburg-Western Pomerania and 48 in Schleswig-Holstein, thus only about one fifth the rate (Fig. 3).

The special evaluation of the StBA for the ELSA study clearly shows an uneven distribution of abortions among the reporting facilities in the federal states. For the entire federal territory, the results show that 7.3% of all reporting facilities (*n* = 84) performed more than 250 abortions in 2020. These “large reporting facilities” performed a total of 47.2% of all abortions nationwide in 2020. It shows that there are major differences between the federal states in the distribution of abortions among large reporting facilities: In Bavaria, they cover 76.9% of abortions, in Hesse 62.4% and in North Rhine-Westphalia 60.9%. In Lower Saxony, on the other hand, they cover 29.5%, and in Mecklenburg-Western Pomerania there is not even a single large reporting facility. Unfortunately, these differentiated data are not available for eight federal states due to the aggregation of size classes by the StBA.

**Fig. 4:**
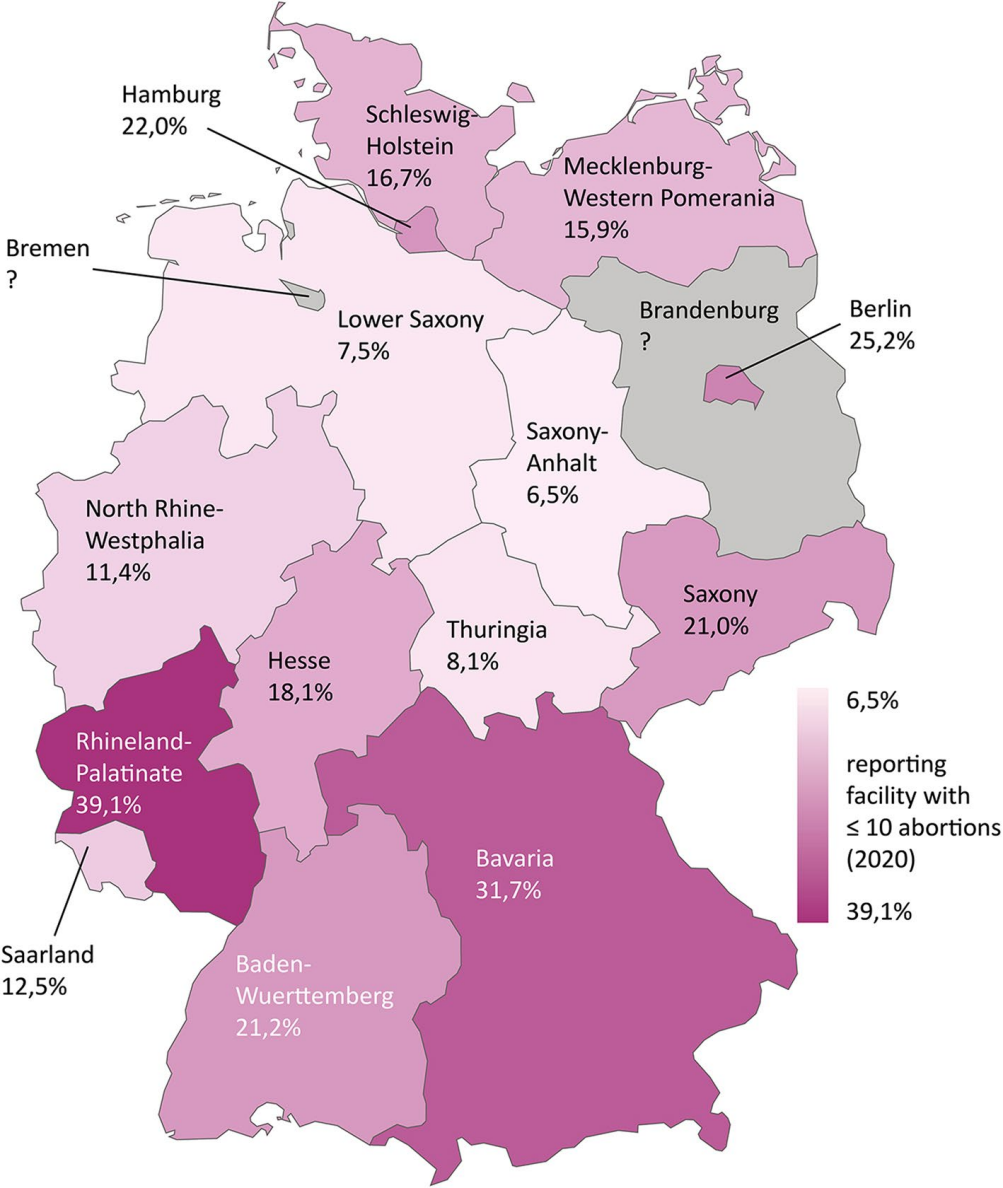
Reporting facilities with 0–10 abortions in Germany (2020)

In contrast, 6.8% of reporting facilities (*n* = 78) provided no abortions and 16.5% of reporting facilities (*n* = 190) only provided 1–10 abortions in 2020. Thus, nearly a quarter of all reporting facilities (23.3%) performed no resp. very few abortions. Here, too, there are large differences between the federal states with regard to the proportion of these “small reporting facilities” among all reporting facilities. The values are highest in Rhineland-Palatinate with 39.1% and Bavaria with 31.7% and lowest in Saxony-Anhalt with 6.5%, Lower Saxony with 7.5% and Thuringia with 8.1% (Fig. 4). Unfortunately, for Bremen and Brandenburg data on the number of small reporting facilities are not available due to the StBA’s aggregation of size classes.

**Fig. 5:**
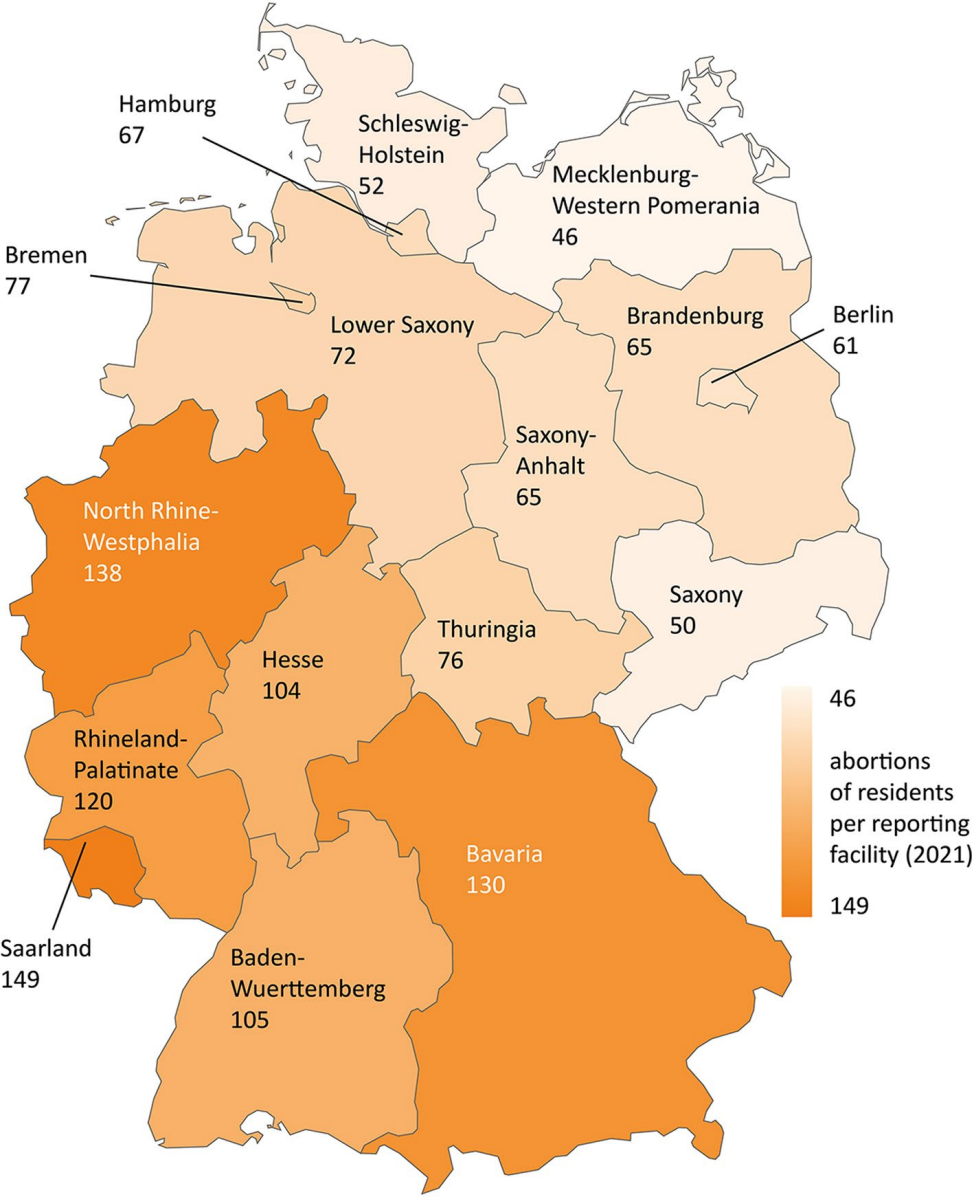
Abortions of residents per reporting facility in Germany (2021)

The figures mentioned are based on the abortions performed in the federal states. For the provision of care, the number of abortions of resident women of the federal states can also be used. This approach would be based on the thesis that the federal states must provide enough services for its resident women. In fact, the abortion rates of women living in the respective federal states differ considerably. The data of the StBA show in detail, how many abortions in one federal state are performed on women living in another federal state or abroad. It also shows, from which origin federal states the women are travelling. For example, 886 of 2220 abortions in Bremen were performed on women living in Lower Saxony in 2021 [18]. Federal states that heavily co-supply patients from other federal states show lower figures in these calculations. These include in particular Saarland (149 compared to 219), Bremen (77 compared to 123) and Hamburg (67 compared to 79). Federal states that are strongly co-supplied by other federal states show correspondingly higher figures here. These include in particular Rhineland-Palatinate (120 instead of 101), Lower Saxony (72 instead of 63) and Baden-Wuerttemberg (105 instead of 95). Nevertheless, there can be seen similar disparities between the eastern and western as well as the northern and southern federal states. The following figure shows the abortions of resident women per reporting facility in the federal states (Fig. 5).

#### Spatial accessibility

**Fig. 6:**
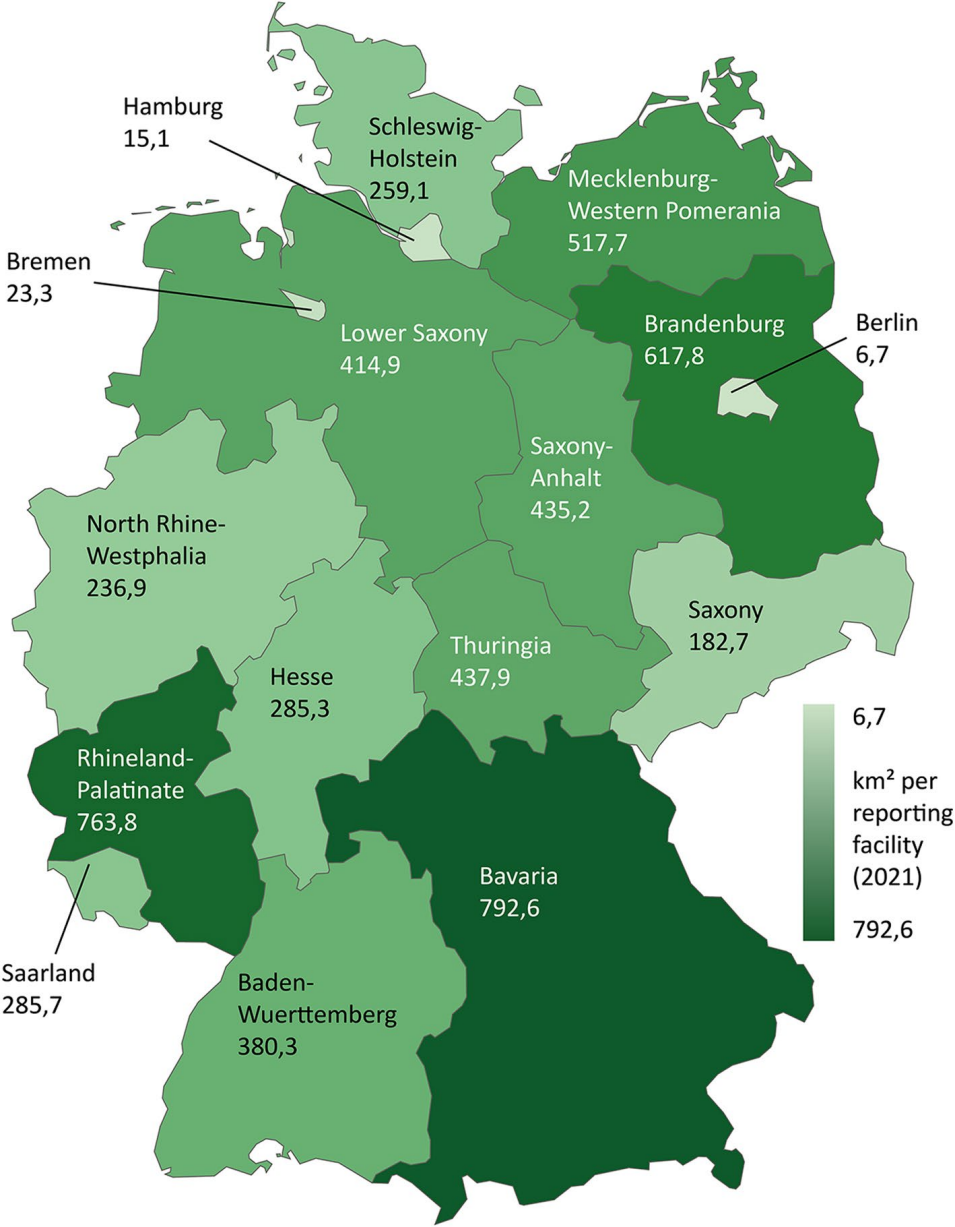
Spatial accessibility in Germany (2021)

The average geographic distribution of reporting facilities varies widely between states as well. The city states of Berlin, Hamburg and Bremen have by far the lowest values (6.7, 15.1 and 23.3 km^2^ per reporting facility). Within the group of territorial states, there are large differences ranging from 182.7 km^2^ per reporting facility (Saxony) to 763.8 km^2^ (Rhineland-Palatinate) resp. 792.6 km^2^ (Bavaria) (Fig. 6).

**Fig. 7:**
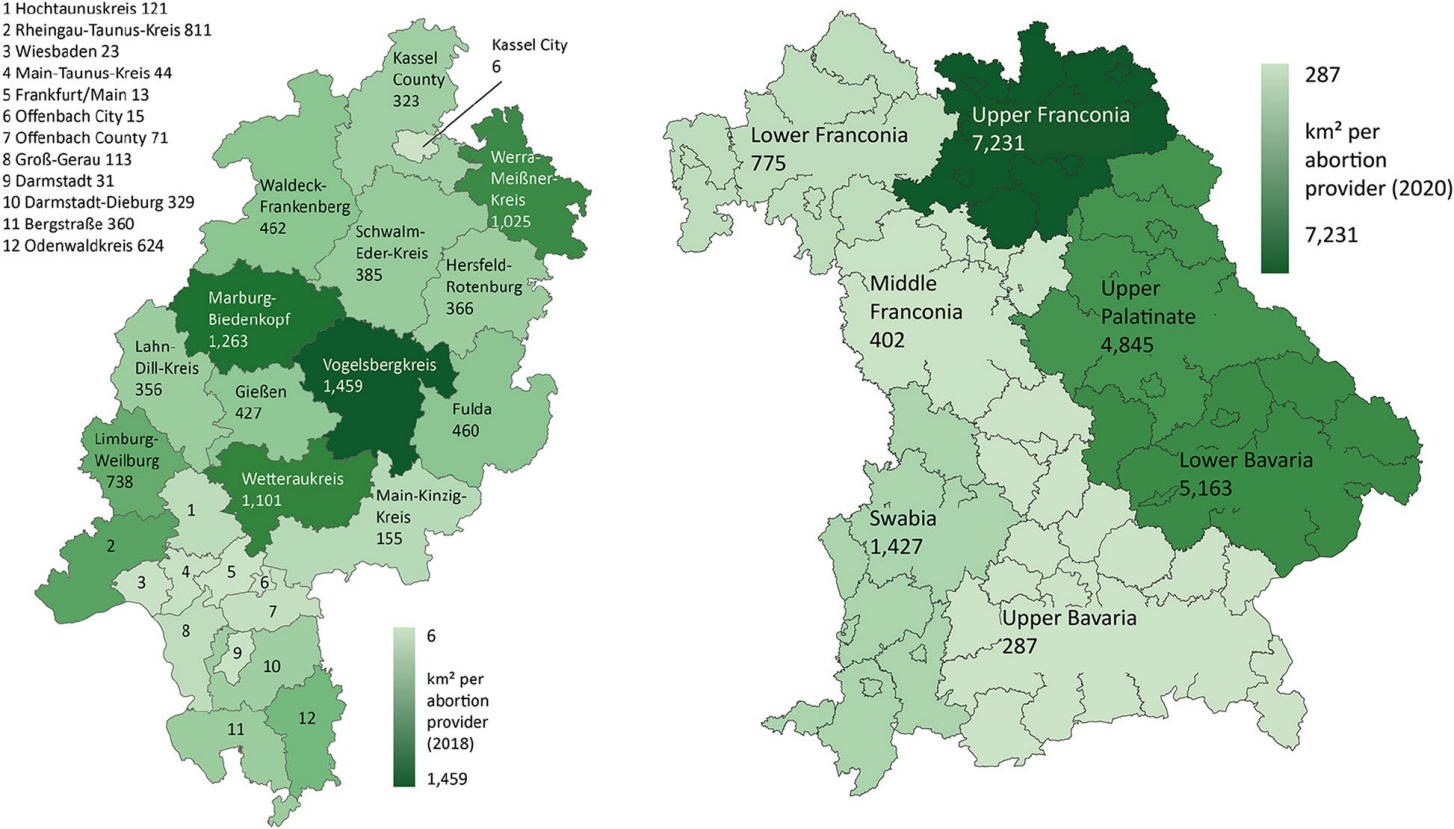
Spatial accessibility by county in Hesse (2018, left) and by administrative district in Bavaria (2020, right)

The examples of Hesse and Bavaria show that there are still much greater regional differences in spatial accessibility within a single federal state. For example, the values for the territorial districts in Hesse range from 44 km^2^ (Main-Taunus district) to over 1459 km^2^ per abortion provider in the Vogelsberg district. For Bavaria, there are also huge regional differences ranging from 287 km^2^ (Upper Bavaria with Munich) to 7231 km^2^ per abortion provider (Upper Franconia) (Fig. 7).

## Discussion

Our analysis of the available data indicates a better availability of abortion providers in the northern and eastern federal states than in the western and southern federal states.

As provided in more detail in chapter 2.1.4, the informative value of the number of reporting facilities is limited. The data does not contain any information about the size of the centre, the capacity for abortions and the days of the week on which they are performed. This information would be highly relevant for the interpretation of regional differences in availability and accessibility of abortion providers. Therefore, our analysis provides answers on tendencies in regional differences.

Provider density is higher in all eastern and northern states (6207 to 13,646 women per reporting facility) than in the western and southern states (17,525 to 30,767 women per reporting facility). The different legal histories in East and West Germany, as well as in the northern and southern states of West Germany, seem to have had an impact on regional differences in abortion provision to this day. One probable reason for the higher provider density in the northern federal states is, that there were more abortions performed before reunification due to the more liberal regulations and the co-supply of women from the southern federal states [21].

The disparity between the northern and eastern states on the one hand and the western and southern states on the other is also evident as a trend in the distribution of abortions among reporting facilities. The figures indicate a more uneven distribution in the western and southern states: In almost all southern and western states more abortions occur on average at one reporting facility (95 to 219 performed abortions per facility). In the northern and eastern states, these numbers are 45 to 123 abortions per facility. Because Bremen provides a high degree of care for patients from other federal states, they show higher figures here. If abortions of residents per facility are compared between the states, the disparities between northern/eastern (46 to 77) and southern/western states (104 to 149) are therefore bigger. This allows the assumption that the lower abortion rates may not compensate for the lower provider density. As we do not have data about the capacity and the size of reporting facilities, it remains speculative, what those differences in provider workload mean in practice for the accessibility and availability of abortion providers for abortion seekers. In addition, a lower abortion rate could also be a consequence of a lower provider density: If women seeking abortions travel abroad or use the services of Women On Web to get an abortion [14], then a lower abortion rate could be also a consequence of a lower provider density. If the regional differences could not be explained by differences in demand, this would be referred to as unjustified differences in healthcare [40].

At the same time, large reporting facilities cover a higher proportion of abortions there, and in some cases the proportion of small reporting facilities among all reporting facilities is also larger. In this context, we assume that an as even as possible distribution of abortions among reporting facilities is particularly less risky with regard to ensuring the availability of abortion providers. If a large proportion of abortions are concentrated at only a few abortion providers, and if provider density is also relatively low, availability may be limited regionally: The loss of a large reporting facility, for example due to retirement, can thus have a particularly negative impact on the regional provision of abortions.

In addition, it can be assumed that a high concentration of abortions at a small number of abortion providers, leads to longer travel distances, which makes it more difficult to reach them geographically and thus to seek care as early as possible, especially in territorial states. Calculations of reporting facilities by area also show large differences between the states and within Bavaria and Hesse. These differences do not (only) depend on the degree of rurality of a region. For example, Schleswig-Holstein, Lower Saxony and Bavaria have similar population densities (168 to 187 people per km^2^) [41] but show large differences in terms of the average spatial accessibility of reporting facilities (259.1, 414.9 resp. 792.6 km^2^ per reporting facility).

It should be noted that calculations of the area per reporting facility say little about real travel times for women seeking to terminate a pregnancy. This is because travel times depend on the transport infrastructure and the means of transport (car, public transport, etc.). Moreover, the figures only represent averages, but the abortion providers are not evenly distributed over the area. In addition, women cross county and state borders due to better accessibility. The figures therefore serve as an initial indication of which regions may have more difficult accessibility. Overall, the states of Rhineland-Palatinate, Baden-Wuerttemberg and Bavaria have both a concentration of abortions in a small number of abortion providers and a low provider density. Rhineland-Palatinate and Bavaria also have the lowest spatial accessibility in comparison.

## Conclusion

In order to evaluate regional availability and accessibility of abortion care, it is necessary to be able to describe the situation quantitatively. Although according to § 13 para. 2 SchKG the federal states are legally obligated to ensure a sufficient number of abortion facilities, there are no further regulations comparable to the needs-based planning of SHI-accredited physicians for the recording and evaluation of the number of abortion facilities. Federal states who do not record data on the number of abortion providers cannot perform their task of ensuring a sufficient number of those facilities as they do not have the data necessary to assess the situation.

It has become clear that the data on abortion services and thus the basis for an adequate assessment of the situation is insufficient, but nevertheless provides indications of regional differences. In order to go beyond a description to an assessment of the care situation, there is a need for clear indicators of when there is good or at least sufficient availability of abortion care services.

First of all, we consider it urgently necessary that the abortion providers in Germany be recorded comprehensively. For this purpose, the number of abortions performed must be recorded. Furthermore, it is necessary to store the data for the chronological course in order to be able to react to declines at an early stage. For all these data, detailed evaluation must be possible, ideally at the county level, but at least differentiated according to spatial planning regions. Furthermore, it is necessary that a desirable ratio between providers and women of reproductive age is defined by law, similar to the regulation on pregnancy counselling centres. Alternatively, abortions could be integrated as services into regular health care, as recommended by the WHO [8], and become components of hospital planning and needs-based planning of SHI-accredited health care.

The ELSA study will use data from our own surveys of physicians, abortion patients, and further special evaluations of the Federal Statistical Office to generate more insights into regional availability and accessibility of abortion care in Germany and to explain regional variations of abortion care.

## Footnotes

1 We refer to people who want to terminate an unwanted pregnancy both in a gender-neutral way as abortion seekers and as women, since most of them see themselves as women and the social stigmatisation of unwanted pregnancy and abortion is linked to gender-specific ideas of femininity, motherhood and the control of female bodies. We want to make these connections visible with the designation. Nevertheless, we point out that people of other genders can also become pregnant unwantedly and thus understand the term in a gender-inclusive way.

2 In Hesse, it was stipulated until 2006 that university hospitals “shall provide an appropriate proportion of gynaecological beds for abortions” (Section 13, Law on the Implementation of Sections 218b and 219 of the Penal Code and Article 3 of the Fifth Law on the Reform of Criminal Law). In the subsequent Hessian Implementation Law on the Conflict of Pregnancy Act (Hessisches Ausführungsgesetz zum Schwangerschaftskonfliktgesetz), such a passage is no longer included. In North Rhine-Westphalia, a similar provision was contained in § 2 (3) of the Hospital Design Act of 11 December 2007. However, in the course of a law to reduce bureaucracy (Gesetz zum Abbau unnötiger und belastender Vorschriften im Land Nordrhein-Westfalen), the paragraph was repealed in 2018.

3 The objective of the ELSA study is to gain insights into the social and health-related stressors and resources of women who have an unwanted pregnant and who carry this pregnancy out or terminate it. Therefore, also the availability, utilization and effectiveness of professional medical support will be analysed. This analysis will be combined with an examination of the current state of and causes for regional diversity within medical support and care services.

4 Germany is divided in 294 counties and 106 district-free cities, which do not belong to any county. Most of them have between 100,000 and 200,000 inhabitants. As population density differs, there are counties with an area bigger than the federal state of Saarland.

5 The size class “0” includes the so-called misreports that result from the regulations on mandatory reporting. Since a reporting facility is obliged to report for 2 years, reporting facilities that have not carried out an abortion in a reporting year are also recorded as reporting facilities.

6 In some federal states, doctors need a separate permit to perform abortions.
